# A delay deviation tolerance IP geolocation method with error estimation

**DOI:** 10.1038/s41598-022-18140-9

**Published:** 2022-08-17

**Authors:** Shuodi Zu, Xiangyang Luo, Shaoyong Du, Liang Wang

**Affiliations:** 1 State Key Laboratory of Mathematical Engineering and Advanced Computing, Zhengzhou, 450001 China; 2 National Digital Switching System Engineering Technology Research Center, Zhengzhou, 450001 China

**Keywords:** Computer science, Information technology

## Abstract

IP geolocation is an important basis of location-based network services, while error estimation is an important basis for judging the reliability of results. Most of the existing IP geolocation algorithms cannot estimate the geolocation error. A few can achieve error estimation through high-precision delay measurement, but their performance is also affected by the common delay inflation in the actual network. A new IP target location estimation method is proposed in this manuscript to achieve geolocation with reliable error estimation of IP targets in actual network. Firstly, after the landmark set divided into training set and verification set for path detection, the metropolitan area network (MAN) topology is extracted through train path set. Secondly, the governed landmarks are searched level by level through the MAN, and the minimum covering circles are calculated through the geographical distribution of the landmarks to infer the routers’ area center. Then, geolocation errors are counted after simulated geolocation through the verification path set, and the minimum mean square error radius of the error mean and the minimum covering circle radius is calculated as the router area radius. Finally, the path to the IP target is measured and compared with the MAN to get the location estimation result. The experimental results based on 12 cities in China show that compared with the existing typical algorithms, the proposed method not only improves the error estimation accuracy, but also has finer geolocation granularity and lower median error.

## Introduction

With the development of network, personalized service is sought after. Obtaining user’s location to provide customized services for users is an important basis of personalized services. Compared with geolocation based on GNSS (Global Navigation Satellite System), IP geolocation technology has lower geolocation accuracy. However, it can provide location-based services not rely on user device assistance, and is widely used in advertising, social networks and network security^1,2^.

Network measurement based IP geolocation is a research hotspot. This kind of method measures the transmission delay and topology information between tshe probe source and the target, combines with the geographical location of the probe source or landmark, and finally estimates target’s location by delay comparison or path matching^3^. Landmark, specific IP whose geographical location is known and unchanged, plays an important role in such geolocation methods. According to the geolocation granularity, it can be divided into two categories: city-level geolocation and street-level geolocation.

City-level geolocation can obtain the regional city-level geographical location of the target IP. Typical geolocation methods include CBG^4^, LBG^5^ and RNBG^6^. CBG^4^ constructs the linear conversion relationship between transmission delay and geographical distance, then forms distance constraints on the target from multiple detection sources to estimate the possible location of the target. After establishing a probability distribution model according to the distance between the detection sources and the landmarks through measurement data, LBG^5^ uses the trained model to locate the target to the area with the greatest probability. RNBG^6^ estimates the location of the target by analyzing the important nodes in the path from detection sources to the target IP, which has strong robustness under different network conditions. This kind of geolocation method can meet the application needs of some Internet service providers(ISP). It is also the basis of street-level geolocation.

Street-level geolocation can obtain higher precision target location estimation, while it also needs a large number of more complex measurement data. The typical geolocation methods include SLG^7^, NNG^8^ and ETBG^9^. SLG^7^ first locates the target to a coarse-grained area based on the improved CBG^4^ algorithm, then continuously adds landmarks in the area, and takes the landmark position with the minimum relative delay with the target as the target’s estimated position. NNG^8^ locates the target to a large area based on the radial basis function network, and then trains the multi-layer perceptron network with the data collected from the area to obtain the fine-grained geographical location of the target. ETBG^9^ processes the landmark set to obtain three data sets, carries out simulated geolocation training based on these data sets, and locates the target according to the MAN obtained from training. This kind of methods has a high geolocation accuracy, but the geolocation performance will also be greatly reduced if the detection data collection is limited, such as delay inflation, route anonymity and so on.

Most geolocation algorithms do not have the ability to estimate the error range of single geolocation results, hard to ensure the reliability of results. For a few algorithms with this capability, high-precision delay measurement is the key means to realize error estimation. However, greatly increasing packet transmission delay caused by service priority and network congestion is widely exists in actual networks, which we usually call it delay inflation. Delay inflation makes it difficult to accurately measure transmission delay and to achieve reliable IP geolocation.

For street-level geolocation, in order to reduce the impact of the above factors on the geolocation results and further improve the performance of geolocation and error estimation, a delay deviation tolerance IP geolocation method with error estimation is proposed in this manuscript. This method not only further improves the geolocation accuracy, but also improves the accuracy of error estimation.

The key contributions of this manuscript are as follows:

1. A new geolocation method is designed. In this method, the IP target is geolocated by calculating the minimum coverage circle corresponding to the landmark distribution. This method predicts the geographic geolocation error through geolocation simulation of multiple data sets. The designed method avoids the influence of delay inflation on the implementation of the method, and further improves the ability of geolocation and error estimation.
2. A target IP location estimation algorithm based on minimum covering circle is proposed. After extracting the metropolitan area network (MAN) topology of the target city, this algorithm calculates the minimum coverage circle corresponding to each node in MAN, and realizes the geographical location estimation of the connected targets of each node.
3. A target IP location error estimation algorithm based on minimum mean square error (MSE) principle is proposed. After using another data set to count the geolocation error of each node in MAN, the algorithm realizes the error estimation of each node by calculating MSE.
4. IP target location experiments are carried out in 12 large cities in different regions in China. The experimental results of the proposed method show that compared with the existing typical location algorithms SLG, RNBG and ETBG, while maintaining the same high success rate of city-level geolocation, the median error of street-level geolocation is reduced by 68.01% and the accuracy of location error estimation is improved by 25.50%.

The rest of this manuscript is organized as follows. In “ETBG’s idea to achieve error estimation” section introduces the operation principle and shortcomings of ETBG which is a typical geolocation algorithm with error estimation. “Method description and analysis” section presents the main framework and the implementation details of the proposed method. “Evaluation” section shows the performance of the method and compares it with typical algorithms, before conclusion in “Conclusion” section .

## ETBG’s idea to achieve error estimation

ETBG is the best IP geolocation algorithm that can estimate error in recent years^9^. Its core idea is to realize the location estimation and error estimation of IP target by estimating the service area of MAN routers.

After obtaining the MAN topology of the target city, ETBG estimates the location of each MAN router. ETBG selects the location from the landmark with the least hops of the router as its location. If there is more than one landmark with the least hops, the location of the landmark with the smallest delay relative to the router is selected as its location. After determining the location of the router, ETBG looks for the farthest landmark in the landmarks connected to the router, and calculates the distance between the farthest landmark and the estimated location of the router as the radius of the service area.

**Figure 1:**
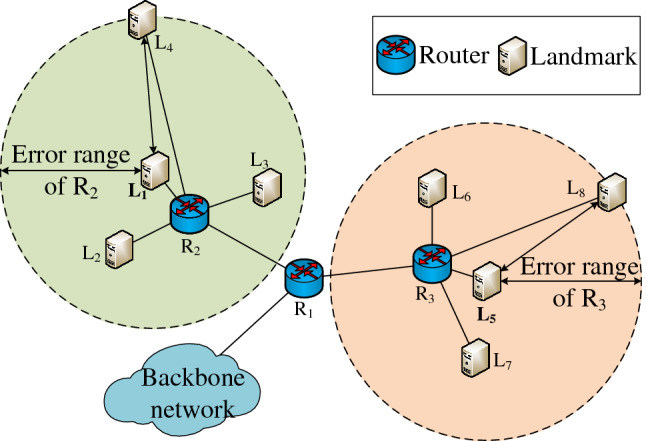
Geolocation schematic diagram of ETBG.

Taking Fig. 1 as an example, after ETBG obtains the landmarks L_1_, L_2_, L_3_ and L_4_ directly connected to router R_2_, it takes the geographical location of L_1_ with the smallest delay as the location of R_2_ by comparing the delay from router to each landmark. Then ETBG calculates the distance between landmarks, and takes the distance between L_1_ and L_4_, which is the farthest from L_1_, as the radius of R_2_’s service area. The service area of R_3_ is obtained by the same principle. As for R_1_, the upper level router of R_2_ and R_3_, ETBG compares the delay between L_1_ and R_2_ with the delay between L_5_ and R_3_, select the smaller one L_1_ as the location of R_1_, and take the distance between L_1_ and L_8_, the farthest one from L_1_, as the radius of R_1_’s service area.

In an ideal network environment, there is a positive correlation between packet transmission delay and geographical distance. Based on this principle, ETBG realizes geolocation and error estimation of IP target. As a result of this, the performance of ETBG is limited by the accuracy of delay measurement.

In the actual network environment, in order to meet the requirements of users for different applications, routers will provide different quality of service for different data streams. The router gives priority to the processing of real-time and important messages. For ordinary messages with weak real-time performance, the processing priority is low, and even discards such messages in case of network congestion. For most routers, the priority of detection packets is low. Work^10^ also points out that in the real network environment, the phenomenon of delay inflation is widespread, which greatly affects the accuracy of delay measurements and the results of geolocation.

When the delay inflation exists, there is no correlation between delay and geographical distance in street-level area. As shown in Fig. 1, if the delay between L_4_ and R_2_ is the smallest, ETBG will take the location of L_4_ as the location of R_2_ and estimate its service area on this basis, resulting in large geolocation error and lower error estimation accuracy.

## Method description and analysis

In this section, after introducing the framework of the proposed method, we describe the four main parts in detail in different subsections.

1. Geolocation framework

Metropolitan area network (MAN) is a broadband local area network established in a city. MAN usually adopts multi-layer network structure to improve security and stability. The high-level router is responsible for the aggregation and distribution of user service data, with a wide service area. The low-level router is mainly responsible for the access and service allocation of users in a specific area^11^. Therefore, after obtaining the geographical distribution of users connected to the router, the geographical area of users in charge of the MAN router can be estimated.

**Figure 2:**
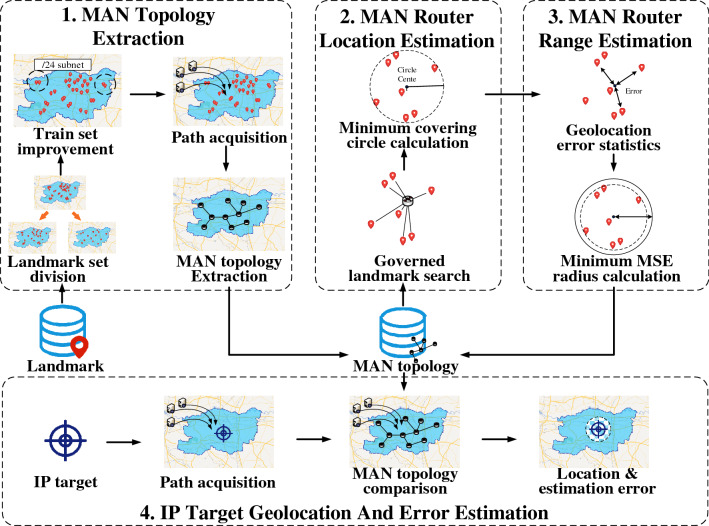
An overview of the IP target location estimation method framework.

According to the layered characteristics of MAN, an IP target location estimation method based on minimum MSE principle is proposed in this manuscript. As shown in Fig. 2, the proposed method consists of the following four parts.

1. *MAN topology extraction.* Divide the landmark set into train set and verification set through bootstrapping sampling, improve the train set by the /24 subnet characteristics, detect the path to the nodes in the train set and verification set, and extract the MAN topology from train path set.
2. *MAN router location estimation.* Search the landmarks governed by MAN routers, and calculate the minimum coverage circles through the geographical distribution of landmarks.
3. *MAN router service area estimation.* Count the geolocation errors of the MAN routers by verification path set, and optimize the radius of the minimum coverage circle by minimum MSE principle.
4. *IP target geolocation and error estimation.* Detect the path to the IP target and compare it with the MAN, determine the geolocation result and error estimation.

These four parts will be elaborated in detail in the following sections.

2. MAN topology extraction

1. Step description

The key of the proposed method is to calculate the geographical distribution characteristics and topological connection between landmarks in the target city. Landmark is an IP node whose geographical location is determined or can be determined. It usually plays the role of reference point in the geolocation process. In this part, path detection is carried out after processing the landmark set data, in order to provide data support for MAN router extraction and topology construction.

This part mainly includes the following four steps.

1. *Landmark set division.* Carry out random sampling with return from landmark set containing N landmarks with N times. Put the selected landmarks into the train set and the unselected landmarks into the verification set.
2. *Train set improvement.* For all /24 subnets involved in the training set, if the number of landmarks in the subnet is greater than 2, select 2 at random; if less than 2, traverse the subnet and add the surviving IP to the training set until the number of landmarks in the subnet is equal to 2 or the traversal ends.
3. *Path acquisition.* Carry out multiple sources detection to detect the path to all nodes in the train set and verification set respectively, remove the backbone nodes and other city nodes according to the delay distribution law, retain the IP nodes belonging to the target city, and obtain the train path set and verification path set respectively.
4. *MAN topology extraction.* Analyze the alias of the train path set, merge different IP nodes corresponding to the same router, sort out the connection relationship between routing nodes in the city, and obtain the MAN topology.

Paper^9^ found that for cities with a small number of landmarks, using the bootstrapping sampling to divide the landmark set will not change the data distribution too much. Since the number of landmarks in the target city cannot be guaranteed, the bootstrapping sampling is used to divide the landmark set. In practical experiments, when cross-city path detection is carried out, the single hop delay in the path will show a "low–high–low" distribution law^10^. Therefore, after getting the detection path, only the part located in the target city is retained according to the change of delay between nodes, so as to reduce the computational burden and error.

After the above steps, two path sets are gotten: train path set and verification path set, and the MAN topology of the target city is further obtained.

(2) Train set improvement

The idea of the proposed method is to obtain the MAN topology of the target city, calculate the service area of each MAN router, and finally estimate the target’s location. The existing mainstream geolocation methods usually detect the landmarks in the target city, analyze the nodes in detection path and obtain the network topology. However, the time required to detect and calculate the topology will be greatly increased if all landmarks are detected. Therefore, the proposed method improves the train set to be detected and retains only a small number of targets for each subnet, which greatly reduces the overhead required for the operation of the method while ensuring the topological integrity.
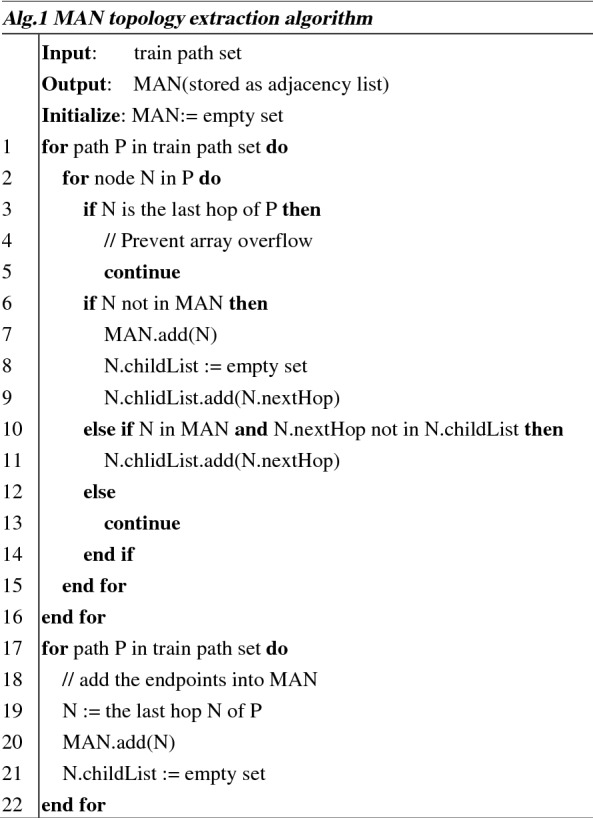


In order to facilitate the configuration and management of the network, ISPs are used to dividing the whole IPs into several subnets and assigning them to different network user groups. It is a common method to divide the IP with the same first 24 bits of IP address into the same subnet, i.e., /24 subnet. In terms of geographical distribution, Mukne found through research that IP in the same /24 subnet tends to be concentrated in the same geographical area^12^. This conclusion is also reached after statistical analysis of landmark data provided by IP2location, IP138, IPIP and other public databases. In terms of topology distribution, the experimental results of Tao based on a total of 46,001 IPs in China show that when one IP of the same /24 subnet is selected for detection, the number of network topology nodes can reach 98% of that of all IPs in the data set^13^. Therefore, in this manuscript, the correlation of /24 subnet nodes in geographical distribution and topological distribution is utilized to find other detectable nodes in the same /24 subnet where the landmark in the train set is located.

For 50,000 city-level landmarks in Hong Kong, 1, 2, 3, 4, and all available IPs were respectively used for each /24 subnet. The topology results are shown in the Table 1.

**Table 1:** Comparison of different IP detection results.

IP number per / 24 subnet	Obtain MAN node points	Obtain MAN node points / probe all IP node points (%)	Time taken (s)	Time spent/time spent detecting all IP (%)
All	1650	100	19,316	100
1	1588	96.24	3567	18.47
2	1636	99.16	4470	23.14
3	1636	99.16	5035	26.07
4	1636	99.16	5621	29.10

It can be seen from the table that when 2 IPs are selected for each/24 subnet to detect, the time is short and enough MAN nodes can be obtained. Therefore, the proposed method improves the train set to take up to 2 detectable IPs per/24 subnet as the rule.

(3) MAN topology extraction

Obtaining MAN topology is the basis of geolocating IP target in this method. Alg.1 describes the implementation of MAN topology extraction. In Alg.1, this manuscript uses adjacency lists to store the MAN topology. The MAN topology is considered as directed graph in which all edges are oriented in the same direction as the message is transmitted during path detection.

In code lines 1 to 16, the algorithm traverses all nodes except the endpoint in the path and adds them to the adjacency list with their next jump. To prevent the array from crossing the bounds during traversal, in code lines 17 to 22, the algorithm adds the endpoints of all paths individually to the adjacency list.

After the MAN topology is obtained, the management area of every MAN router can be estimated by counting the landmarks directly or indirectly connected to these MAN routers.

3. MAN router location estimation

1. Step description

The data message reaches the destination after being forwarded level by level by the MAN routers. The higher the level of MAN router, the more users are responsible. On the contrary, the lower the level, the more users governed by the router tend to gather in a certain area. This part estimates the location and size of these areas through the distribution of landmarks.

This part mainly includes the following two steps.

1. *Governed landmark search.* Traverse the train path set, record the IP of each landmark into the attributes of each MAN router in the path, and mark it as these routers’ governed landmark.
2. *Minimum covering circle calculation.* Traverse the MAN routers, calculate the corresponding minimum covering circle for each router according to the geographical distribution of its governed landmarks, and record the center position and radius in the MAN router attribute.

After the above steps, the minimum covering circle is calculated corresponding to each MAN router through search of governed landmarks. The circle center can be regarded as the center of the MAN router service area.

(2) Calculation of minimum covering circle

How to use the known landmarks to obtain the service area of each MAN router is the key of the proposed method. In MAN, the number and distribution range of users in the charge of different routers vary. In general, the lower a router level is, the fewer users it is responsible for, and therefore the more geographically concentrated those users are. Aiming at this characteristic, the proposed method uses the known landmarks to simulate the geolocation process to obtain the service area of the MAN routers, i.e., its error range.

Two methods were tested to determine the service area. One is to calculate the corresponding minimum covering circle according to the distribution of governed landmarks of the router to obtain the corresponding center and radius. Minimum covering circle is the smallest circle that can cover a group of points on the plane, and was first proposed by Sylvester^14^. The other is to calculate the arithmetic center of the governed landmarks of the router as the service area center, and the distance between the landmark furthest from the arithmetic center and the center itself as the radius.

**Figure 3:**
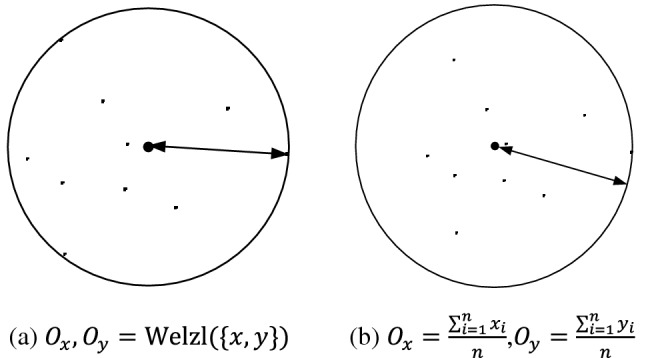
Schematic diagram of two area determination methods.

Figure 3 shows the schematic diagram of two service area determination methods. In Fig. 3, $\{x,y\}$ represents the point set, $({x}_{i},{y}_{i})$ represents the coordinates of each point, and $\left({O}_{x},{O}_{y}\right)$ represents the coordinates of the center of the circle. Figure 3a shows the minimum covering circle based method, while the arighmetic center based method is shown in Fig. 3b.

In order to test the specific performance of the two methods in location estimation, we conducted a comparative experiment of 5442 street-level landmarks in Hong Kong is conducted, and tested the mean error(mean),median error(median),minimum error mean(min), maximum error mean(max) and error estimation accuracy mean(error) under different methods. The experimental results are shown in Table 2. As shown in Table 2, the method based on the minimum covering circle improves the error estimation accuracy by 20% at the cost of reducing the geolocation accuracy by 6%, and the geolocation accuracy is also higher than the existing in^9^. Therefore, this manuscript determines the center and radius of the service area based on the minimum covering circle.

**Table 2:** Comparison of service area estimation methods.

Method	Mean (km)	Median (km)	Min (km)	Max (km)	Error (km)
Minimum covering circle	5.68	5.55	4.09	7.61	3.69
Arithmetic center	5.00	4.78	3.43	7.31	4.40
Paper ^9^	11.16	8.72	–	–	3.97

Alg. 2 describes the specific implementation of this part. In code lines 1 to 18, through traversing the train path set, landmarks connected to nodes in the MAN are counted and recorded in the MAN attributes. In code lines 19 to 24, by traversing each node in MAN, Welzl’s algorithm^15^ is utilized to calculate the center and radius of the corresponding minimum covering circle through the distribution of governed landmarks. In MAN, the governed landmarks of the routers in the higher layer are the union of the governed landmarks of all routers in the lower layer. Welzl’s algorithm is one of the most common methods to calculate such problems. The minimum covering circle problem is included in a class of ordinary linear programming problems, which can be solved by algorithms like Welzl based on linear programming. How to calculate the minimum covering circle is not the focus of this manuscript, so Welzl’s algorithm is directly used in the proposed method.
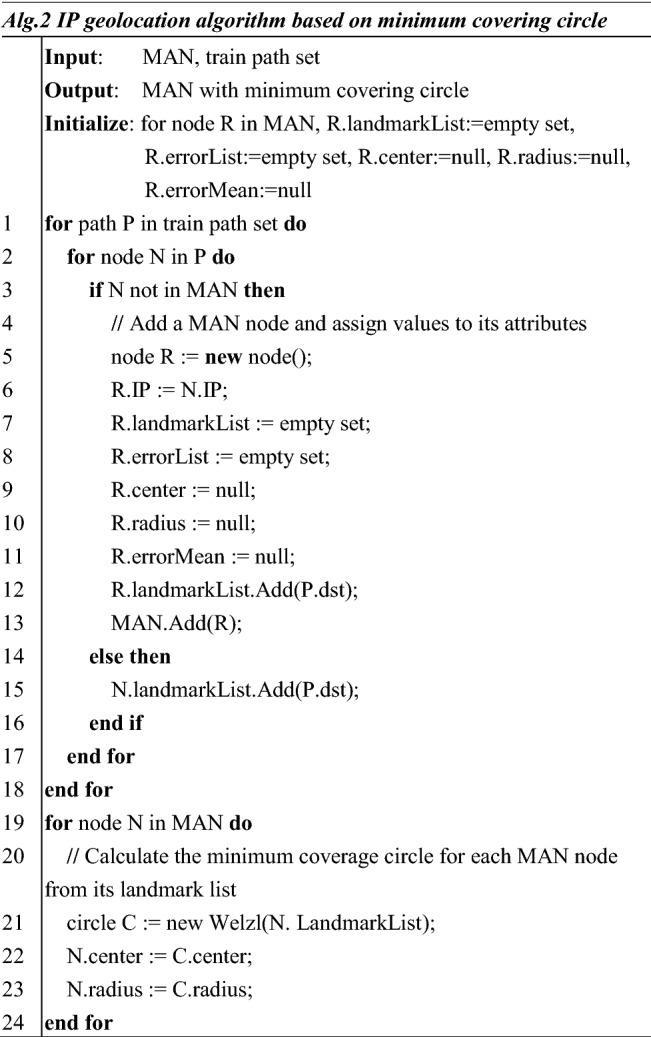


In the actual implementation, we build a MAN router database, in which the fields of each record include the router IP, the list of governed landmarks, as well as the location and radius of service area.

4. MAN router area estimation

1. Step description

In the previous part, the locations of MAN routers’ government area are inferred by searching for governed landmarks and calculating the corresponding minimum covering circle. This part calculates the geolocation error by verification path set, optimizes the radius of the minimum coverage circle by the minimum MSE principle, and estimates the size of the MAN router service area.
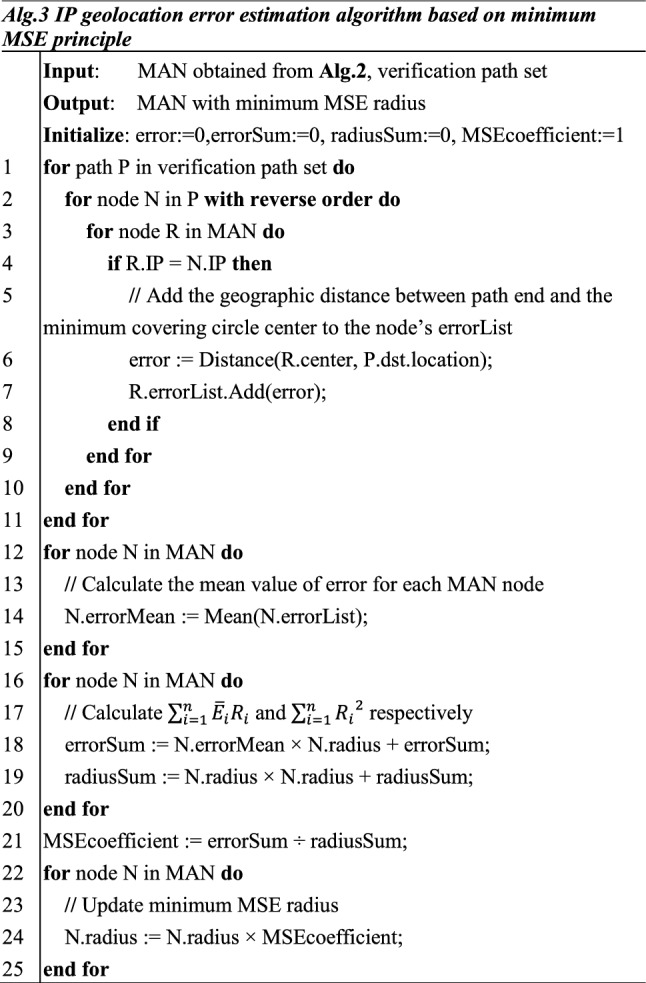


This part mainly includes the following two steps.

1. *Geolocation error statistics.* Traverse the verification path set, and judge whether there is intersection with the MAN for each path. If it exists, select the MAN router with the least hops from the end of the path, calculate the geographical distance between the end of the path and the center of the minimum covering circle corresponding to the router, and add it into the error list of the MAN router as the geolocation error.
2. *Minimum MSE radius calculation.* Traverse the MAN routers. For each router, calculate the mean value ${\overline{E} }_{i}$ of the error list. The radius ${R}_{i}$ has been obtained from the previous part. The minimum MSE radius $m{MSER}_{i}$ can be calculated as below:
  1 $$mMSER_{i} = R_{i} \frac{{\mathop \sum \nolimits_{i = 1}^{n} \overline{E}_{i} R_{i} }}{{\mathop \sum \nolimits_{i = 1}^{n} R_{i}^{2} }}$$

After calculation, record the minimum MSE radius in the MAN router attribute as area estimation.

After the above steps, the attributes of the MAN topology of the target city are further improved, and each router has its own area estimation.

(2) Derivation process of minimum MSE radius

In section ‘MAN router location estimation’ the minimum covering circle is obtained by calculating the distribution of governed landmarks of the routers. Its attributes include center and radius. The center of the circle can be estimated as the location center of the MAN node, while it is not appropriate to use the radius of the minimum covering circle as the radius of the server area. As we all know, the larger the area is, the greater the probability that the IP target is located in the area is. However, increasing the radius also reduces the geolocation accuracy. Therefore, how to make the estimated area as small as possible and at the same time to include as many nodes as possible is an important problem to be solved in this method.

In this manuscript, the minimum MSE principle is utilized to calculate the service area of MAN nodes. For the minimum covering circle radius of all node in the MAN, multiply them by a parameter to get the service area radius under the principle of minimum MSE error.

For each MAN node, its mean error is ${\overline{E} }_{i}$, and the calculated minimum covering circle radius is ${R}_{i}$. The difference between the geolocation error and radius of each MAN node is $|{\overline{E} }_{i}-{R}_{i}|$.

For the MAN, the MSE of the difference between the geolocation error and the minimum covering circle radius is expressed by:

2 $$MSE = \frac{{\mathop \sum \nolimits_{i = 1}^{n} \left( {\overline{E}_{i} - R_{i} } \right)^{2} }}{n}$$

For the convenience of calculation, multiply ${R}_{i}$ by a parameter $a$. Thus there is:

3 $$MSE = \frac{{\mathop \sum \nolimits_{i = 1}^{n} \left( {\overline{E}_{i} - aR_{i} } \right)^{2} }}{n}$$

The variable parameter in (3) is $a$. If MSE is minimized, the derivation of (3) can be obtained as follow:

4 $$\frac{{{\text{d}}MSE}}{{{\text{d}}a}} = \frac{{2*\mathop \sum \nolimits_{i = 1}^{n} \left( {\overline{E}_{i} - aR_{i} } \right)\left( { - R_{i} } \right)}}{n}$$

It is easy to see that when $\sum_{i=1}^{n}\left({\overline{E} }_{i}-a{R}_{i}\right)(-{R}_{i})=0$, the MSE takes the minimum. Then it can be deduced as below:

5 $$\mathop \sum \limits_{i = 1}^{n} \overline{E}_{i} *R_{i} - \mathop \sum \limits_{i = 1}^{n} aR_{i}^{2} = 0$$

6 $$a = \frac{{\mathop \sum \nolimits_{i = 1}^{n} \overline{E}_{i} R_{i} }}{{\mathop \sum \nolimits_{i = 1}^{n} R_{i}^{2} }}$$

Thus, for each MAN node, the service area radius under the principle of minimum MSE could be calculated as follow:

7 $$mMSER_{i} = R_{i} \frac{{\mathop \sum \nolimits_{i = 1}^{n} \overline{E}_{i} R_{i} }}{{\mathop \sum \nolimits_{i = 1}^{n} R_{i}^{2} }}$$

Through the equation above, the area radius under the principle of minimum MSE of each MAN router, i.e., the minimum MSE radius, can be calculated, and then the geolocation error can be estimated. Alg. 3 describes the specific implementation of this part. In code lines 1 to 11, for each path in the verification path set, the minimum coverage circle’s center corresponding to the MAN node with the least hops away from the end of the path is found. Then the geographical distance between the center and the end of the path is calculated as the geolocation error. In code lines 12 to 25, through traversing each node in MAN, the mean value of its error list is calculated as its corresponding radius.

5. IP target geolocation and error estimation

After completing the improvement of the attributes of the MAN router, there are lists of governed landmarks of the MAN routers, the approximate location and scope of service areas of each router. Through this information, the location of the target can be estimated. While detecting the path to the target, the method in paper^10^ is also used in the proposed method to save the part of the path in the target city. The two steps in this part are as follows:

1. *Path acquisition.* Detect the target and get the detection path, remove the nodes belong to backbone network and other city in the path according to the delay distribution, and retain the nodes belonging to the target city.
2. *MAN topology comparison.* Judge whether the path obtained in the previous step intersects with the MAN topology. If it exists, select the MAN router closest to the end of the path, and output its corresponding minimum covering circle center and minimum MSE radius as geolocation result and error estimation.

This method can not only achieve the target location estimation, but also obtain the error estimation of the geolocation result, which will increase the reliability of the result.

## Evaluation

In order to verify the geolocation ability of the proposed method, relevant experiments are carried out and compared with typical geolocation algorithms such as SLG^7^, NNG^8^, RNBG^6^, ETBG^9^.

1. Experimental setup

1. Data set

When we tested the performance of the algorithms, the landmark data used in this manuscript is mainly obtained by the following two ways:

- From public databases: From the existing public databases, the IPs with street-level location in the query return result are evaluated by the method in paper^16^, and the IP addresses with reliable location are reserved.
- From Internet data mining: Using the methods in paper^17^ and paper^18^, the landmarks are obtained through Internet yellow pages and web maps, and associated with the actual geographical locations, and then the reliability is evaluated by the method in paper^16^, so as to retain the IP address with reliable location.

After mining, screening and evaluation of landmarks, the scale of landmarks in the experimental cities in this paper is shown in Table 3. Each landmark data contains longitude, latitude and IP.

**Table 3:** Number of landmarks in experimental cities.

City	Beijing	Chengdu	Changsha	Fuzhou	Guangzhou	Hong Kong	Hangzhou	Nanjing	Shanghai	Taiyuan	Wuhan	Zhengzhou
Number	408,719	102,350	56,204	37,936	98,564	122,445	169,990	84,726	414,382	78,254	125,327	69,814

(2) Evaluation index

We utilized city-level geolocation capability, street-level geolocation capability and error estimation capability to judge the proposed method’s performance. These three indexes are described in detail as follows.

- Success rate of city-level geolocation ${R}_{s}$

The success rate of city-level geolocation is the ratio of the number of successfully located IPs to the total number of located IPs. It is defined as follow:

8 $$R_{s} = \frac{{C_{s} }}{{C_{t} }}$$

In (8), ${C}_{s}$ is the number of valid and correct geolocation results returned by the algorithm in the geolocation process, and ${C}_{t}$ is the total number of geolocated IPs. This index is also adopted by paper^6^ and paper^9^.

(b) Street-level geolocation error ${E}_{mean},{E}_{median}$

Street-level geolocation error is the distance between the geographical location returned by the algorithm and target’s real geographical location when geolocating the IP target with known location. It is defined as below:

9 $$E_{geo} = \sqrt {\left( {Geo_{lon} - Site_{lon} } \right)^{2} + \left( {Geo_{lat} - Site_{lat} } \right)^{2} }$$

In (9), ${Geo}_{lon}$ and ${Geo}_{lat}$ are the longitude and latitude of the IP target to be located returned by the geolocation algorithm, and ${Site}_{lon}$ and ${Site}_{lat}$ are the longitude and latitude of the actual geographical location of the IP target to be located. Because the street-level geolocation area is small and the calculation error between plane coordinates and spherical coordinates can be ignored, the plane coordinate system is directly used to calculate the distance between two points in the proposed method. After a large number of geolocation experiments, the mean ${E}_{mean}$ and median ${E}_{median}$ of geolocation error can be calculated as evaluation indexes. This index is used in most street level geolocation algorithms, including paper^7^, paper^8^ and paper^9^.

(iii) *Geolocation* error estimation accuracy ${P}_{e}(k)$

The estimation ability of street-level geolocation error is determined by the difference between the actual geolocation error corresponding to a single geolocation and the estimation error returned by the geolocation algorithm. It is defined as follows:

10 $$D_{e} = \left| {E_{geo} - E_{est} } \right|$$

11 $$P_{e} \left( k \right) = \frac{{|\{ x \in D_{e} |x < k\} |}}{{\left| {\left\{ {D_{e} } \right\}} \right|}}$$

${E}_{geo}$ is the actual error in a single geolocation, ${E}_{est}$ is the estimation error returned by the geolocation algorithm, and $\{{D}_{e}\}$ is the set of difference values between the two errors. After a large number of geolocation experiments, ${P}_{e}(5)$ and ${P}_{e}\left(10\right)$, the proportion of difference less than 5 km and 10 km in the total number, were counted as the evaluation indexes. This index appears in paper^9^, and we have made a specific formula expression for it in this manuscript.

(3) Detection mode

The devices among different ISPs in China are independent and the network stratification is obvious. CBG has poor geolocation performance in such network environment. Therefore, when testing SLG, we provided landmarks in small areas, and only used its fine-grained geolocation method. NNG uses neural network for geolocation, and can't give the name of the city, so we didn’t carry out city-level geolocation experiments on it.

In this manuscript, 4 probe sources in China were deployed. Scamper^19^ developed by CAIDA to initiate path detection for the target is utilized in this manuscript. In addition, when detecting the topology information of the target network, this manuscript comprehensively uses five types of protocols: ICMP, TCP, UDP, ICMP-Paris and UDP-Paris, and improves the acquisition scale of topology information by using multi-protocol path detection. ICMP-Paris and UDP-Paris can also avoid the generation of wrong path information^20^.

(4) Comparison method

In this manuscript, SLG^7^, RNBG^6^, NNG^8^ and ETBG^9^ are used as comparison methods. The detailed configuration is shown in the Table 4.

**Table 4:** Experimental setup.

Experiment	Detection source distribution	Probe message type	Experimental accuracy	Comparison method
City-level geolocation	Zhengzhou Shenzhen Beijing Chengdu	ICMP TCP UDP ICMP-paris UDP-paris	City-level	SLG^7^ RNBG^6^ ETBG^9^ Proposed method
Street-level geolocation			Street-level	SLG^7^ NNG^8^ ETBG^9^ Proposed method
Geolocation error evaluation				ETBG^9^ Proposed method

2. Analysis of experimental results
3. City-level geolocation capability

**Figure 4:**
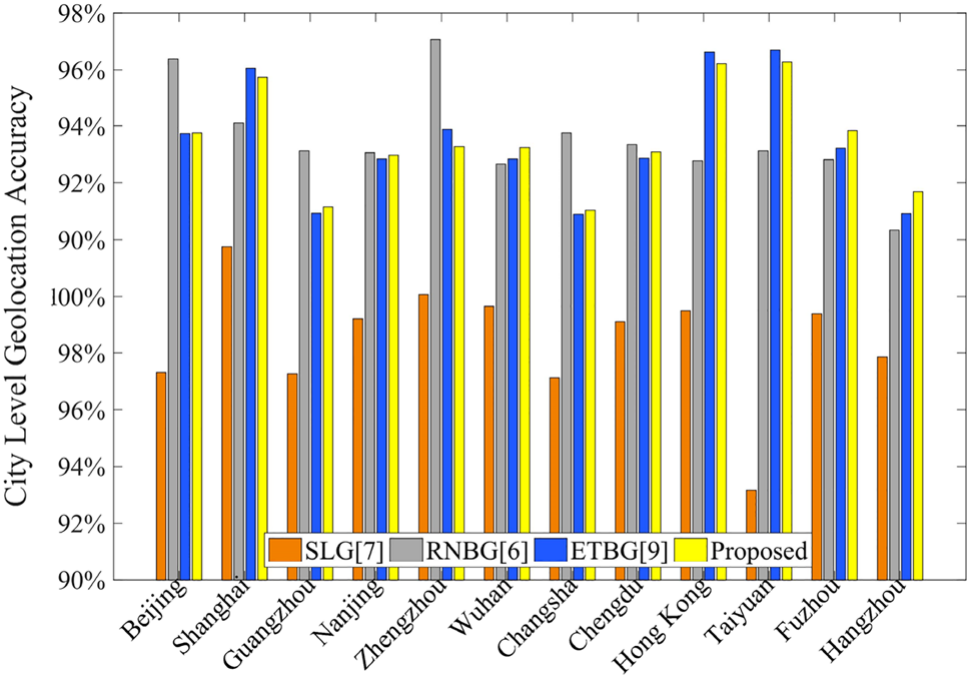
Comparison of city-level geolocation capability.

In order to ensure the most basic city-level geolocation ability, 12 cities in China is selected in this manuscript to conduct geolocation experiments under the same ISP. The experimental results are shown in Fig. 4.

According to statistics, in the same ISP environment, the city-level geolocation success rate of SLG algorithm is 94.23%, that of RNBG is 97.78%, that of ETBG is 97.73%, and that of the proposed method is 97.76%. Compared with SLG, the proposed method has a higher success rate of city-level geolocation. While it has the similar city-level geolocation ability with RNBG, the proposed method can estimate the location of targets with higher accuracy like ETBG.

(2) Street-level geolocation capability

**Figure 5:**
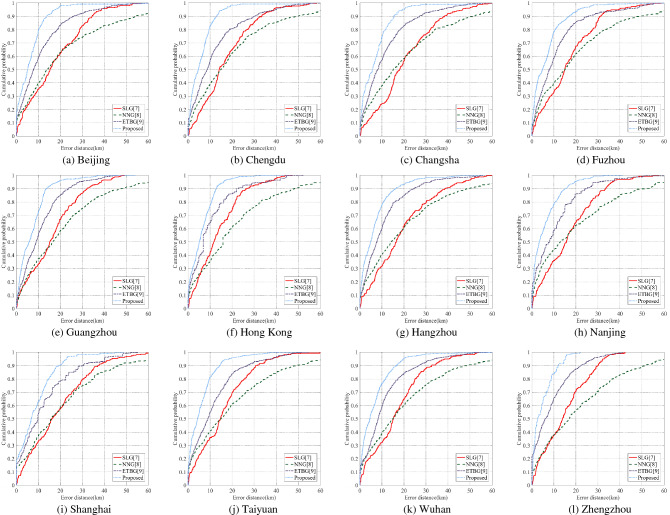
Comparison of street-level geolocation capability.

Street-level geolocation experiments were carried out in the above cities. Figure 5 shows the cumulative error probability of geolocation experiment, i.e., the proportion of results less than a given geolocation error to all geolocation results. In Fig. 5, the closer the curve is to the upper left corner, the smaller the geolocation error is. According to statistics, the mean geolocation errors of SLG, NNG, ETBG and the proposed method are 16.81 km, 20.77 km, 10.82 km and 6.58 km, and the median errors are 15.12 km, 14.43 km, 7.91 km and 4.83 km. We can see that the geolocation results of the proposed method are better than SLG, NNG and ETBG.

(3) Geolocation error evaluation capability

**Figure 6:**
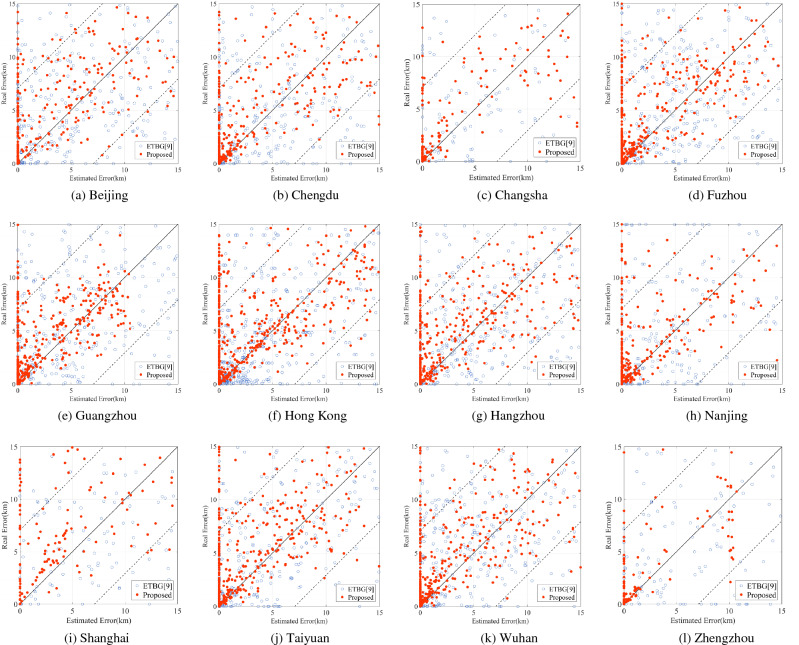
Street-level geolocation error scatter plotss.

On the basis of street-level geolocation, the error estimation comparison experiment is carried out in the above cities. The results are shown in Table 5 and Fig. 6.

**Table 5:** Geolocation error estimation capability comparison.

Method	Min (km)	Max (km)	Mean (km)	Median (km)	${{\varvec{P}}}_{{\varvec{e}}}(5)$(%)	${{\varvec{P}}}_{{\varvec{e}}}(10)$(%)
ETBG	0	63.66	4.29	1.87	62.73	78.82
Proposed method	0	66.74	3.68	1.38	76.31	90.29

In Fig. 6, each point represents a geolocation result. The distance from the point to the horizontal axis represents the geolocation accuracy. In Fig. 6, the closer the distance from the point to the horizontal axis is, the higher the geolocation accuracy is. The distance from the point to the angle bisector of the two coordinate axes represents the error estimation accuracy. The closer the distance from the point to the angle bisector is, the higher the error estimation accuracy is. The geolocation results with a difference of no more than 5 km between the estimated error and the actual error are located between the two dotted lines in the figures. We can see that most of the geolocation results are maintained at a low error level.

When the number of landmarks in the target city is not enough and cannot spread over enough subnets, some routers in the extracted MAN will be connected to only one landmark. In this case, if this router is used for geolocation, the estimation error may be equal to 0 and the actual error may be greater than 0, corresponding to the points on the y-axis in Fig. 6. The proposed method and ETBG both have such problems. When the number of landmarks connected to the router is greater than 1, the actual error of ETBG may be equal to 0 and the estimation error may be greater than 0, corresponding to the points on the x-axis.

According to statistics, compared with ETBG, the proportion of the difference between the estimated error and the actual error less than 5 km increased from 62.73 to 76.31%, and the proportion of the difference less than 10 km increased from 78.82 to 90.29%.

(4) Geolocation capability summary

For comparison, the results of different types of geolcoation experiments and error estimation experiment are summarized in Table 6. As shown in Table 6, compared with typical algorithms such as SLG, NNG, RNBG and ETBG, the proposed method further improves the geolocation ability. The city-level geolocation success rate is improved to 97.72%, the street-level geolocation median error is reduced to 4.78 km, and the error estimation capability is improved by more than 11.57%.

**Table 6:** Geolocation capability summary.

Method	SLG^7^	NNG^8^	RNBG^6^	ETBG^9^	Proposed method
${\mathrm{R}}_{\mathrm{s}}$(%)	94.23	–	97.78	97.73	97.76
Geolocation ability in city	Y	Y	N	Y	Y
${\mathrm{E}}_{\mathrm{mean}}$(km)	16.81	20.77	–	10.82	6.58
${\mathrm{E}}_{\mathrm{median}}$(km)	15.12	14.43	–	7.91	4.83
Error estimation capability	N	N	N	Y	Y
${\mathrm{P}}_{\mathrm{e}}(5)$(%)	–	–	–	62.73	78.72
${\mathrm{P}}_{\mathrm{e}}(10)$(%)	–	–	–	76.31	90.29

## Conclusion

To solve the problem that the geolocation performance and error estimation accuracy of existing IP geolocation algorithms are reduced in the delay inflation environment, a delay deviation tolerance IP geolocation method with error estimation is presented in this manuscript. This method divides the data set and carries out path measurement respectively, extracts the MAN topology of the target city, estimates the routers’ location based on the landmark distribution, estimates the router service area through simulated geolocation, and finally realizes the target location estimation and error estimation through path detection and comparison with the MAN. Compared with the existing typical algorithms, this method not only has higher error estimation accuracy, but also has better geolocation granularity and lower geolocation error.

## Data Availability

The datasets generated and analyzed during the current study are not publicly available due to the security of network facilities and the privacy of user location, but are available from the corresponding author on reasonable request. Meanwhile, the original landmark data used in this manuscript is available at: https://www.ipip.net/ and https://www.ipplus360.com/.
